# Diverting phenylpropanoid pathway flux from sinapine to produce industrially useful 4-vinyl derivatives of hydroxycinnamic acids in Brassicaceous oilseeds

**DOI:** 10.1016/j.ymben.2022.01.016

**Published:** 2022-03

**Authors:** Guillaume N. Menard, Mollie Langdon, Rupam Kumar Bhunia, Aishwarya R. Shankhapal, Clarice Noleto-Dias, Charlotte Lomax, Jane L. Ward, Smita Kurup, Peter J. Eastmond

**Affiliations:** a Department of Plant Science, Rothamsted Research, Harpenden, Hertfordshire, AL5 2JQ, UK; b National Agri-Food Biotechnology Institute, Mohali, Punjab, 140306, India; c Department of Computational and Analytical Sciences, Rothamsted Research, Harpenden, Hertfordshire, AL5 2JQ, UK

**Keywords:** Metabolic engineering, Plant, Oilseed, 4-Vinyl phenol, 4-Vinyl guaiacol, 4-Vinylsyringol

## Abstract

Sinapine (sinapoylcholine) is an antinutritive phenolic compound that can account for up to 2% of seed weight in brassicaceous oilseed crops and reduces the suitability of their protein-rich seed meal for use as animal feed. Sinapine biosynthesis draws on hydroxycinnamic acid precursors produced by the phenylpropanoid pathway. The 4-vinyl derivatives of several hydroxycinnamic acids have industrial applications. For example, 4-vinyl phenol (4-hydroxystyrene) is a building block for a range of synthetic polymers applied in resins, inks, elastomers, and coatings. Here we have expressed a modified bacterial phenolic acid decarboxylase (PAD) in developing seed of *Camelina sativa* to redirect phenylpropanoid pathway flux from sinapine biosynthesis to the production of 4-vinyl phenols. PAD expression led to a ∼95% reduction in sinapine content in seeds of both glasshouse and field grown *C. sativa* and to an accumulation of 4-vinyl derivatives of hydroxycinnamic acids, primarily as glycosides. The most prevalent aglycone was 4-vinyl phenol, but 4-vinyl guaiacol, 6-hydroxy-4-vinyl guaiacol and 4-vinylsyringol (Canolol) were also detected. The molar quantity of 4-vinyl phenol glycosides was more than twice that of sinapine in wild type seeds. PAD expression was not associated with an adverse effect on seed yield, harvest index, seed morphology, storage oil content or germination in either glasshouse or field experiments. Our data show that expression of PAD in brassicaceous oilseeds can supress sinapine accumulation, diverting phenylpropanoid pathway flux into 4-vinyl phenol derivatives, thereby also providing a non-petrochemical source of this class of industrial chemicals.

## Highlights

• A phenolic acid decarboxylase was expressed in developing *Camelina sativa* seeds.
• Production of the antinutritive phenolic compound sinapine was reduced by 95%.
• Hydroxycinnamic acids were converted to 4-vinyl phenols and accumulated as glycosides.
• The quantity of 4-vinyl phenols was more than twice that of sinapine in wild type.
• Seed yield appeared not to be affected in either glasshouse or field experiments.

## 1 Introduction

Brassicaceous plants accumulate significant amounts of sinapoyl esters, which are derived from a branch of the phenylpropanoid pathway (Fraser and Chapple, 2011). In oilseed rape (*Brassica napus* L.), sinapoylcholine (sinapine) accumulates predominantly in the developing embryo and can account for up to 2% of the dry weight of the seed (Baumert et al., 2005)|. Upon seed germination, the sinapine is metabolised to sinapoylmalate, which is stored in the cotyledons of the developing seedling (Lorenzen et al., 1996). Sinapoylmalate, along with other phenolics, has been ascribed a function in protecting aerial tissues against ultraviolet B radiation (Kusano et al., 2011; Landry et al., 1995; Meiβner et al., 2008). Unfortunately, the presence of sinapoyl esters in rapeseed, and other Brassicaceous oilseeds, reduces the suitability of their protein-rich seed meal for use as animal feed. Sinapine has a bitter taste and reduces protein digestibility (Shahidi and Naczk, 1992). To improve the nutritional value of the seed meal, it is considered necessary to reduce the sinapoyl ester content to below 2 mg g^-1^ of dry weight. Natural variation in sinapine content ranges from 3.4 to 12.9 mg g^-1^ in rapeseed (Zum Felde et al., 2007) and so several studies have used targeted genetic modiﬁcation of metabolic pathway genes to lower sinapine levels (Harloff et al., 2012).

**Fig. 1:**
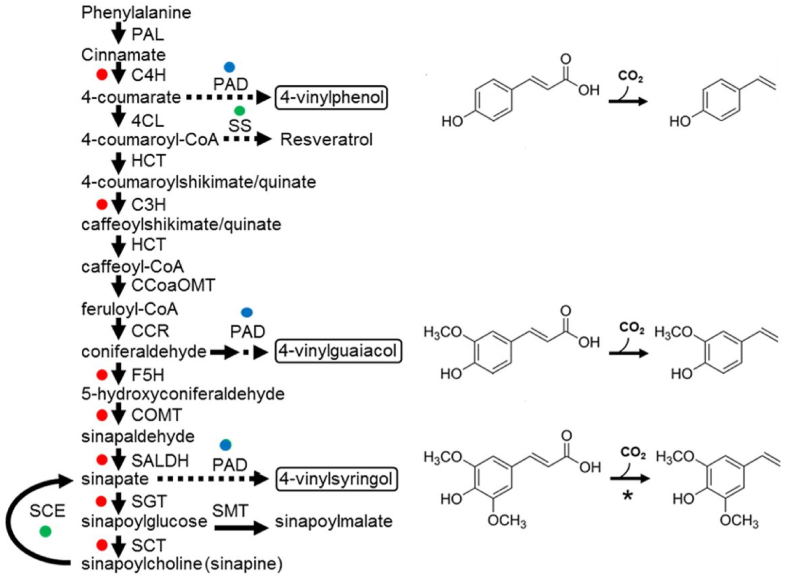
A schematic diagram of the main pathway of sinapoyl ester biosynthesis in Brassicaceous seeds. The diagram is adapted from Milkowski and Strack (2010). Red dots mark enzymatic steps that have previously been suppressed, and green dots enhanced, leading to a reduction in sinapine content. Predicted products of heterologous PAD expression in this study (blue dots) are highlighted in boxes and the corresponding reactions and chemical structures are depicted on the right. Asterisk denotes the reaction that can only be catalysed by modified PAD (Morley et al., 2013). SS, stilbene synthase; PAD, phenolic acid decarboxylase; PAL, phenylalanine-ammonia lyase; C4H, cinnamate-4-hydroxylase; 4CL, 4-coumarate:CoA ligase; HCT, hydroxycinnamoyl-CoA:shikimate/quinate hydroxycinnamoyltransferase; C3H, 4-coumarate ester 3-hydroxylase; CCoAOMT, caffeoyl-CoA O-methyltransferase; CCR, cinnamoyl-CoA reductase; F5H, ferulate 5-hydroxylase; COMT caffeate O-methyltransferase; SALDH, sinapaldehyde dehydrogenase; SGT, UDP-glucose:sinapate glucosyltransferase; SCT, sinapoylglucose:choline sinapoyltransferase; SCE, sinapine esterase; SMT, sinapoylglucose:malate sinapoyltransferase. (For interpretation of the references to colour in this figure legend, the reader is referred to the Web version of this article.)

To date, seven genes encoding enzymes of sinapine biosynthesis have been disrupted in rapeseed, either individually or in combination, using gene silencing or mutagenesis (Fig. 1; (Bhinu et al., 2009; Emrani et al., 2015; Hüsken et al., 2005; Mittasch et al., 2013; Nair et al., 2000; Weier et al., 2007). These genes encode cinnamate-4-hydroxylase (C4H), 4-coumarate ester-3-hydroxylase (C3H), caffeate O-methyltransferase (COMT), ferulic acid 5-hydroxylase (FAH), sinapaldehyde/coniferaldehyde dehydrogenase (SALDH), UDP-glucose:sinapate glucosyltransferase (SGT) and sinapoylglucose:choline sinapoyltransferase (SCT). SGT catalyses the committed step of sinapoyl ester biosynthesis (Fig. 1) and its disruption has been reported to have the largest individual effect, reducing sinapine content by up to 71% and total sinapoyl ester content by up to 76% (Emrani et al., 2015; Wolfram et al., 2010). Clauβ et al. (2011) have also overexpressed sinapine esterase (SCE) in rapeseed, depleting seed sinapine content by up to 95% (Fig. 1). However, this method does not reduce the levels of the remaining sinapoyl esters and the seeds also exhibit some unexpected biochemical and morphological changes (Clauβ et al., 2011).

An alternative approach to reduce the sinapoyl ester content in Brassicaceous seeds is to redirect the flux of hydroxycinnamic acid precursors into other, more beneficial, products. Hüsken et al. (2005) have previously shown that 4-coumaroyl-CoA can be diverted to produce resveratrol glucoside (piceid) in rapeseed SGT RNA-interference lines by expressing a stilbene synthase from grapevine (*Vitis vinifera*) (Fig. 1). Resveratrol (3,5,4′-trihydroxy-*trans*-stilbene) is commonly used in dietary supplements and has been claimed to have antioxidant, anti-inflammatory, cardioprotective and anti-cancer properties (Hüsken et al., 2005). Interestingly, hydroxycinnamic acids can be harmful to bacteria and some possess a detoxification pathway that employs a cofactor-free phenolic acid decarboxylase (PAD) enzyme (Tran et al., 2008). PADs can produce a range of 4-vinyl phenols (4-VPs) with industrial applications (Huccetogullari et al., 2019) (Fig. 1). 4-vinyl phenol (4-VP), 4-vinyl guaiacol (4-VG) and 4-vinylsyringol (4-VS) are all natural flavour and aroma compounds (Vanbeneden et al., 2008) and 4-VG, in particular, is used as an ingredient in the food, perfume and cosmetic industries because of its clove-like taste and aroma (Li et al., 2019; Yang et al., 2009). 4-VS (Canolol) is a natural food preservative, owing to its potent antioxidant properties (Matthäus et al., 2014; Wakamatsu et al., 2005). 4-VP (4-hydroxystyrene) also serves as a building block for a range of synthetic polymers applied in resins, inks, elastomers, and coatings (Qi et al., 2007). Poly(4-VP) (PVP) is particularly important in electronics, where it is used as a photoresist and as a dielectric layer in organic transistors; for example of organic thin-film-transistor liquid–crystal displays (Qi et al., 2007). 4-VP is currently produced from petroleum-based feedstock and 4-VS and 4-VG by the respective chemical decarboxylation of ferulic and sinapic acids (Li et al., 2019). Several studies have explored more sustainable production routes for 4-VPs based on fermentation of genetically engineered microorganisms (Qi et al., 2007; Verhoef et al., 2009; Huccetogullari et al., 2019; Li et al., 2019; Robinson et al., 2020), but none have tried to engineer plants to make 4-VPs. To our knowledge, 4-VS is the only vinyl derivative of a 4-hydroxycinnamic acid that has previously been reported in angiosperms, where low levels (>50 μg g^-1^) are found in rapeseed and are presumed to be the product of spontaneous thermal decarboxylation of sinapine (Matthäus et al., 2014; Wakamatsu et al., 2005). 4-VP glucosides have also been detected in the ferns *Pteridium aquilinum* and *Asplenium trichomanes* (Ojika et al., 1987; Revoltella et al., 2019).

The aim of this study was to determine whether the flux of hydroxycinnamic acid intermediates in Brassicaceous oilseeds can be diverted to produce 4-VPs, instead of antinutritional sinapoyl esters (Fig. 1). We envisage that the 4-VPs could then be extracted from the seed as sustainable value-added coproducts of oilseed processing. Around 70 million metric tons of rapeseeds are processed each year (https://www.fas.usda.gov/data/oilseeds-world-markets-and-trade). Cofactor-free PAD genes have been identified in over 100 bacteria (Rodriguez et al., 2021) and several have been cloned and characterised, including from *Bacillus pumilus* strain UI-670 (Zago et al., 1995). Although this enzyme is active on a range of 4-hydroxycinnamic acids, including 4-coumaric and ferulic acids (Huang et al., 1994; Matte et al., 2010), it does not accept sinapic acid as a substrate (Morley et al., 2013). Morley et al., (2013) successfully extended *B. pumilus* PAD substrate specificity to include sinapic acid, using knowledge of the X-ray crystal structure (Matte et al., 2010) combined with saturation mutagenesis of the active site. Substitution of isoleucine 85 for alanine (I85A) resulted in sinapic acid decarboxylase activity that is ∼60% of that with ferulic acid (Morley et al., 2013). We therefore decided to study the effect of seed-specific overexpression of this modified I85A version of *B. pumilus* PAD (Morley et al., 2013) in *Camelina sativa* (L.) Cranz. *C. sativa* is a Brassicaceous oilseed crop that is being developed as a platform for the production of a range of higher value food and non-food products (Malik et al., 2018). *C. sativa* is highly amenable to genetic engineering (Nguyen et al., 2013) and is known to contain sinapoyl esters in its seed meal (Matthäus and Zubr, 2000).

## 2 Materials and methods

### 2.1 Plant material and growth conditions

*Camelina sativa* (cv. Suneson) plants were grown in l L pots containing Levington F1 compost in a glasshouse set to a 16 h light (23 °C)/8 h dark (16 °C) period, with supplemental light provided when ambient levels fell below 400 μmol m^2^ s^1^. The field experiment was conducted at Rothamsted Research (Harpenden, Hertfordshire, U.K.; grid reference TL120130) under UK Department for Environment, Food & Rural Affairs consent 19/R08/01 (consent holder: Professor Johnathan Napier). The field trial site consisted of multiple small experimental plots separated by a 0.5 m unsown break and surrounded by a 6 m wide WT *C. sativa* guard, which served as a “buffer” to mitigate the dispersal of pollen. Two 1.2 by 4.2 m plots were allocated for each *ProGLY:PAD* line, plus WT controls. The trial plots were machine planted in spring 2019 at a depth of 2 cm and a sowing density of 300 plants m^2^. The plots were protected by horticultural fleece until the seedlings had emerged and were subsequently irrigated and manually weeded, as necessary. At maturity, 1 m^2^ sections of the plots were harvested manually by cutting at the base of the stand. The material was transported to the containment glasshouse facility to dry fully before threshing and weighing of seed and straw to determine yield and harvest index.

### 2.2 Cloning and plant transformation

A modified *B. pumilus* PAD gene carrying an I85A substitution (Morley et al., 2013) was codon optimised for expression in *C. sativa* and synthetized by Genewiz (Supplementary Fig. 1). The open reading frame was then amplified by PCR using Q5 TAQ polymerase (NEB) and primers PAD-F and PAD-R (Supplementary Table 1). The PCR product was purified using a gel extraction kit (Promega) and digested using EcoR1 and Xho1 (Promega). pBinGlyRed2 (Nguyen et al., 2013) was also digested with EcoRI and Xho1, alkaline phosphatase treated (Promega), gel-purified and PAD was ligated into the vector using T4 DNA ligase (NEB). Transgenic *C. sativa* lines were then generated as previously described (Ruiz-Lopez et al., 2014). The vector was transformed into *Agrobacterium tumefaciens* strain AGL1. *C. sativa* inflorescences were immersed in the Agrobacterium suspension for 30 s without applying any vacuum. Seeds harvested from transformed plants were screened for expression of the DsRed marker protein using a Leica M205 fluorescence stereo microscope fitted with a DsRed filter.

### 2.3 Gene expression analysis

For each sample, around 50 mg of seeds were ground in liquid nitrogen using a pestle and mortar. The Qiagen Plant RNeasy kit (with RLC buffer) was used to extract RNA following the manufacturer's protocol. The Superscript III kit (Invitrogen) was used to produce the cDNA. cDNA samples were normalised and quantitative PCR was perform on a Roche LightCycler 96 using the FastStart Essential DNA Green Master mix (Roche) with the following condition: preincubation for 10 min at 95 °C, 45 cycles of three steps amplification (95 °C for 10s, 60 °C for 15s, 72 °C for 15s), melting was performed from 65 °C to 97 °C. The primer pairs are listed in Supplementary Table 1 (PAD-F1 and PAD-R1). Two *C. sativa* reference genes UbOxRed1 and Actin-2 were selected from Chao et al. (2019). Data were analysed using the LighCycler 96 software and Qbase^+^ (Biogazelle).

### 2.4 Soluble metabolite extraction and quantification

For initial metabolite profiling by NMR and UHPLC-MS triplicate aliquots of 20 seeds from each sample were homogenised using a vibration mill (Retsch MM300 with Qiagen rack adaptors for microcentrifuge tubes). Two tungsten carbide beads (3 mm) were used per samples and milling was carried out at 30 Hz for 5 min at room temperature. Samples were suspended in either CD_3_OD/D_2_O (80:20 v/v, 1 mL) + 0.01% TSP for ^1^H-NMR or CH_3_OH/H_2_O (80:20 v/v, 1 mL) for UHPLC-MS. Samples were extracted in the vibration mill for 5 min and then heated at 50 °C (10 min). After centrifugation (13200 rpm, 10 min), extracts were transferred to glass vials for UHPLC-MS and to 5 mm NMR tubes for analysis.

^1^H-NMR spectra were collected using a Bruker Avance 600 MHz NMR spectrometer (Bruker Biospin) operating at 600.05 MHz (^1^H). Spectra were acquired at 300 K using a 5 mm TCI cryoprobe using 16 scans and by using the zgpr pulse sequence with a 90° angle. Residual water was suppressed by pre-saturation during a 5 s delay. Spectra consisted of 64,000 data points and a spectral width of 12 ppm. FIDs were automatically transformed within Topspin v2.1 (exponential window and a line broadening of 0.5 Hz). Phasing and baseline correction were carried out within the instrument software and chemical shifts were referenced and quantified relative to an internal standard (d4-TSP, 0.01% w/v). Quantitation of sinapine and choline were achieved via integration of the peaks appearing at δ3.283–3.265 and δ3.232–3.213 respectively. Total 4-VP derivatives were quantified via the integration of peaks appearing between δ6.735–6.615.

UHPLC–MS were recorded on an LTQ-Orbitrap Elite mass spectrometer coupled to a Dionex UltiMate 3000 RS UHPLC system (Fisher Scientific). Samples were injected (10 μL) onto a reversed-phase Hypersil GOLD C18 selectivity HPLC column (3 μm, 30 × 2.1 mm i.d. Thermo Fisher Scientific) maintained at 35 °C. The solvent system consisted of water/0.1% formic acid (A) and acetonitrile/0.1% formic acid (B). Total run time was 40 min using a flow rate of 0.3 mL/min and the following elution gradient: 0–5 min, 0% B; 5–27 min, 31.6% B; 27–34 min, 45% B; 34–37.5 min,75% B. Mass spectra were collected using a heated ESI source and mass spectra were acquired in negative mode with a resolution of 120,000 over m/z50–1500. The source voltage, sheath gas, auxiliary gas, sweep gas and capillary temperature were set to 2.5 kV, 35 (arbitrary units), 10 (arbitrary units), 0.0 (arbitrary units) and 350 °C, respectively. Default values were used for other acquisition parameters. Automatic MS–MS fragmentation was performed on top four ions using an isolation width of m/z 2. Ions were fragmented using high-energy C-trap dissociation with a normalised collision energy of 65 and an activation time of 0.1 ms. Data was collected and inspected using Xcalibur v. 2.2 (Thermo Fisher Scientific). Data were analysed using Compound discoverer (Thermo Fisher Scientific). Raw data files were uploaded and samples were categorised according to their transgenic event. A standard workflow (Untargeted metabolomics) was utilised to perform retention time alignment, unknown compound detection and elemental composition prediction. Data was processed with an intensity threshold of 100,000. The resultant data table was analysed within compound discoverer (Principal Component Analysis) to determine initial clustering and was then subjected to a differential analysis to reduce the data table to statistically significant up- and down-regulated metabolite features. Metabolites were identified by comparison to characterised isolated standards and by inspection of characteristic fragmentation patterns.

### 2.5 Compound isolation and structural characterisation

For compound isolation, seeds from transgenic line R30-3-2 (500 mg) were homogenised and extracted with H_2_O/CH_3_OH (80:20 v/v, 2 mL), as described in section 2.4. Samples were heated to 50 °C (10 min) in a water bath. After centrifugation, the supernatant was placed in a glass HPLC vial for fractionation and isolation. Compounds were isolated via repeated injection onto a reversed-phase Ascentis C18 column (5 μm, 5 × 250 mm, Supelco). General experimental procedures for HPLC, NMR spectroscopy and UHPLC-MS have been reported previously (Noleto-Dias et al., 2018).

*3,4,5-trihydroxystyrene-diglucopyranoside*: UHLPC-MS: RT 16.68 min; *m/z* 475.1451 [M-H]^-^ calc'd for C_20_H_28_O_13_. ^1^H NMR (D_2_O:CD_3_OD, 4:1) δ 6.92 (1H, d, *J* = 1.8 Hz, H-6), 6.82 (1H, d, *J* = 1.8 Hz, H-2), 6.66 (1H, dd, *J* = 10.8, 17.7 Hz, H-7), 5.76 (1H, d, *J* = 17.7 Hz, H-8), 5.29 (1H, d, *J* = 10.8 Hz, H-8), 5.11 and 5.00 (2H, d, *J* = 7.7 Hz, H-1′ and H-1″), 4.00–3.23 (overlapping sugar signals).

*4-vinylphenol-diglucopyranoside*: UHLPC-MS: RT 18.91 min; *m/z* 443.1553 [M-H]^-^ calc'd for C_20_H_28_O_11_. ^1^H NMR (D_2_O:CD_3_OD = 4:1) δ 7.48 (2H, d, *J* = 8.7 Hz, H-2/6), 7.10 (2H, d, *J* = 8.7 Hz, H-3/5), 6.76 (1H, dd, *J* = 11.1, 17.7 Hz, H-7), 5.75 (1H, dd, *J* = 0.7, 17.7 Hz, H-8), 5.23 (1H, d, *J* = 0.7, 11.0 Hz, H-8), 5.32 (d, *J* = 7.3 Hz, H-1′ and H-1″), 4.00–3.23 (overlapping sugar signals).

*3,4,5-trihydroxystyrene-(malonyl)-diglucopyranoside*: UHLPC-MS: RT 19.21 min; *m/z* 561.1457 [M-H]^-^ calc'd for C_23_H_30_O_16_. ^1^H NMR (D_2_O:CD_3_OD = 4:1) δ 6.90 (1H, d, *J* = 1.8 Hz, H-6), 6.81 (1H, d, *J* = 1.8 Hz, H-2), 6.66 (1H, dd, *J* = 10.9, 17.7 Hz, H-7), 5.76 (1H, d, *J* = 17.6 Hz, H-8), 5.29 (1H, d, *J* = 11.0 Hz, H-8), 5.10 and 4.99 (2H, d, *J* = 7.6 Hz, H-1′ and H-1″), 4.00–3.23 (overlapping sugar signals).

*4-vinylphenol-rhamnopyranosyl-glucopyranoside*: UHLPC-MS: RT 20.70 min; *m/z* 427.1601 [M-H]^-^ calc'd for C_20_H_28_O_10_. ^1^H NMR (D_2_O:CD_3_OD = 4:1) δ 7.49 (2H, d, *J* = 8.8 Hz, H-2/6), 7.10 (2H, d, *J* = 8.8 Hz, H-3/5), 6.76 (1H, dd, *J* = 11.0, 17.7 Hz, H-7), 5.76 (1H, dd, *J* = 0.6, 17.7 Hz, H-8), 5.24 (1H, dd, *J* = 0.6, 11.0 Hz, H-8), 5.08 (1H, d, *J* = 7.5 Hz, H-1′), 3.56 (1H, dd, *J* = 7.6, 8.8 Hz, H-2′), 3.70 (1H, m, H-3′), 3.51 (1H, t, *J* = 9.0, H-4′), 3.59 (1H, m, H-5′), 4.02 (1H, dd, *J* = 1.2, 10.7 Hz, H-6′), 3.70 (1H, m, H-6′), 4.76 (1H, d, *J* = 1.4 Hz, H-1″), 3.91 (1H, dd, *J* = 1.4, 3.4 Hz, H-2″), 3.77 (1H, dd, *J* = 3.4, 9.7 Hz, H-3″), 3.40 (1H, t, *J* = 9.7 Hz, H-4″), 3.71 (1H, dd, *J* = 9.7, 6.2 Hz, H-5″), 1.20 (3H, d, *J* = 6.2 Hz, H-6″).

*4-vinylphenol-apiofuranosyl-glucopyranoside*: UHLPC-MS: RT 20.83 min; *m/z* 413.1439 [M-H]^-^ calc'd for C_19_H_26_O_10_. ^1^H NMR (D_2_O:CD_3_OD = 4:1) δ 7.49 (2H, d, *J* = 8.8 Hz, H-2/6), 7.09 (2H, d, *J* = 8.8 Hz, H-3/5), 6.76 (1H, dd, *J* = 11.0, 17.7 Hz, H-7), 5.75 (1H, dd, *J* = 0.6, 17.7 Hz, H-8), 5.24 (1H, dd, *J* = 0.6, 11.0 Hz, H-8), 5.18 (1H, d, *J* = 7.7 Hz, H-1′), 3.75–3.50 (4H, m, H-2′/3′/4′/5′), 3.92 (1H, dd, *J* = 2.1, 12.6 Hz, H-6′), 3.75 (1H, dd, *J* = 5.8, 12.6 Hz, H-6′), 5.41 (1H, d, *J* = 2.3 Hz, H-1″), 4.02 (1H, d, *J* = 2.3 Hz, H-2″), 4.02 (1H, d, *J* = 10.0 Hz, H-4″), 3.87 (1H, d, *J* = 10.0 Hz, H-4″), 3.59 (2H, s, H-5″).

*4-vinylguaiacol-rhamnopyranosyl-glucopyranoside*: UHLPC-MS: RT 21.47 min; *m/z* 503.1763 [M + formate-H]^-^ calc'd for C_22_H_32_O_13_. ^1^H NMR (D_2_O:CD_3_OD = 4:1) δ 7.22 (1H, d, *J* = 1.8 Hz, H-2), 7.13 (1H, d, *J* = 8.4 Hz, H-5), 7.09 (1H, dd, *J* = 1.8, 8.4 Hz, H-6), 6.74 (1H, dd, *J* = 11.0, 17.7 Hz, H-7), 5.78 (1H, d, *J* = 17.7 Hz, H-8), 5.26 (1H, d, *J* = 11.0 Hz, H-8), 3.90 (3H, s, 3-OC**H**_**3**_), 5.07 (1H, d, *J* = 7.4 Hz, H-1′), 3.60 (1H, dd, *J* = 7.4, 8.1 Hz, H-2′), 3.68 (1H, m, H-3′), 3.51 (1H, t, *J* = 9.0 Hz, H-4′), 3.59 (1H, m, H-5′), 3.99 (1H, dd, *J* = 1.2, 10.7 Hz, H-6′), 3.70 (1H, m, H-6′), 4.75 (1H, d, *J* = 1.5 Hz, H-1″), 3.89 (1H, dd, *J* = 1.7, 3.4 Hz, H-2″), 3.75 (1H, dd, *J* = 3.4, 9.7 Hz, H-3″), 3.39 (1H, t, *J* = 9.6 Hz, H-4″), 3.68 (1H, m, H-5″), 1.16 (3H, d, *J* = 6.2 Hz, H-6″). ^13^C NMR (D_2_O:CD_3_OD = 4:1) δ 136.4 (C-1), 113.2 (C-2), 152.2 (C-3), 148.4 (C-4), 119.3 (C-5), 122.7 (C-6), 139 (C-7), 116.5 (C-8), 58.9(3-O**C**H_3_), 103.5 (C-1′), 76.0 (C-2′), 78.0 (C-3′), 72.5 (C-4′), 78.8 (C-5′), 69.3 (C-6′), 103.4 (C-1″), 73.2 (C-2″), 73.3 (C-3″), 75.2 (C-4″), 71.6 (C-5″), 19.4 (C-6″).

*4-vinylphenol-(5ʹ-sulfonyl, 6ʹ-malonyl)-glucopyranoside*: UHLPC-MS: RT 23.11 min; *m/z* 447.0599 [M-H]^-^ calc'd for C_17_H_20_O_12_S. ^1^H NMR (D_2_O:CD_3_OD = 4:1) δ 7.50 (2H, d, *J* = 8.8 Hz, H-2/6), 7.09 (2H, d, *J* = 8.8 Hz, H-3/5), 6.75 (1H, dd, *J* = 11.0, 17.7 Hz, H-7), 5.76 (1H, d, *J* = 17.7 Hz, H-8), 5.23 (1H, d, *J* = 11.0 Hz, H-8), 5.13 (1H, d, *J* = 7.9 Hz, H-1′), 3.67 (1H, m, H-2′), 3.75 (1H, m, H-3′), 4.27 dd (1H, dd, *J* = 9.0, 9.5 Hz, H-4′), 4.02 (1H, ddd, *J* = 2.2, 6.0, 9.5 Hz, H-5′), 4.57 (1H, dd, *J* = 2.4, 12.4 Hz, H-6′), 4.34 (1H, dd, *J* = 6.0, 12.4 Hz, H-6′).

*4-vinylphenol-apiofuranosyl-(6′-malonyl)-glucopyranoside*: UHLPC-MS: RT 23.88 min; *m/z* 499.1458 [M-H]^-^ calc'd for C_22_H_28_O_13_. ^1^H NMR (D_2_O:CD_3_OD = 4:1) δ 7.49 (2H, d, *J* = 8.8 Hz, H-2/6), 7.07 (2H, d, *J* = 8.8 Hz, H-3/5), 6.76 (1H, dd, *J* = 11.0, 17.6 Hz, H-7), 5.76 (1H, d, *J* = 17.7 Hz, H-8), 5.23 (1H, d, *J* = 11.0 Hz, H-8), 5.18 (1H, d, *J* = 7.5 Hz, H-1′), 3.75–3.50 (4H, m, H-2′/3′/4′/5′), 4.49 (1H, dd, *J* = 2.2, 12.5 Hz, H-6′), 4.34 (1H, dd, *J* = 6.2, 12.5 Hz, H-6′), 5.41 (1H, d, *J* = 2.2 Hz, H-1″), 4.02 (1H, d, *J* = 2.2 Hz, H-2″), 4.02 (1H, d, *J* = 10.0 Hz, H-4″), 3.87 (1H, d, *J* = 10.0 Hz, H-4″), 3.59 (2H, s, H-5″).

*4-Vinylphenol-(sulfonyl, malonyl)-rhamnopyranosyl-glucopyranoside:* UHLPC-MS: RT 23.47 min; *m/z* 593.1171 [M-H]^-^ calc'd for C_23_H_30_O_16_S. ^1^H NMR (600 MHz, D_2_O-CD_3_OD) δ 7.50 (2H, d, *J* = 8.8 Hz, H-2/6), 7.06 (2H, d, *J* = 8.8 Hz, H-3/5), 6.76 (1H, dd, *J* = 17.5, 10.9 Hz, H-7), 5.76 (1H, d, *J* = 17.7 Hz, H-8), 5.28 (1H, d, *J* = 7.7 Hz, H-1ʹ), 5.23 (1H, d, *J* = 11.0 Hz, H-8), 5.19 (1H, d, *J* = 1.3 Hz, H-1ʹʹ), 1.28 (3H, d, *J* = 6.3 Hz, H-6ʹʹ), 4.00–3.23 (overlapping sugar signals).

*4-vinylphenol-rhamnopyranosyl-(6′-malonyl)-glucopyranoside*: UHLPC-MS: RT 24.32 min; *m/z* 513.1611 [M-H]^-^ calc'd for C_23_H_30_O_13_. ^1^H NMR (D_2_O:CD_3_OD = 4:1) δ 7.50 (2H, d, *J* = 8.8 Hz, H-2/6), 7.06 (2H, d, *J* = 8.8 Hz, H-3/5), 6.75 (1H, dd, *J* = 10.9, 17.7 Hz, H-7), 5.76 (1H, dd, *J* = 0.7, 17.7 Hz, H-8), 5.23 (1H, dd, *J* = 0.7, 10.9 Hz, H-8), 5.27 (1H, d, *J* = 7.4 Hz, H-1′), 3.69 (2H, m, H-2′/3′), 3.56 (1H, d, *J* = 9.5 Hz, H-4′), 3.83 (1H, ddd, *J* = 2.2, 5.8, 9.5 Hz, H-5′), 4.47 (1H, dd, *J* = 2.2, 12.2 Hz, H-6′), 4.32 (1H, dd, *J* = 5.8, 12.2 Hz, H-6′), 5.17 (1H, d, *J* = 1.7 Hz, H-1″), 4.05 (1H, dd, *J* = 1.8, 3.3 Hz, H-2″), 3.69 (1H, m, H-3″), 3.42 (1H, m, H-4″), 3.90 (1H, dd, *J* = 9.6, 6.3 Hz, H-5″), 1.26 (3H, d, *J* = 6.3 Hz, H-6″).

### 2.6 Lipid analysis

Seed oil and moisture contents of whole seeds were measured by low-resolution time domain NMR spectroscopy using a Minispec MQ20 device (Bruker) fitted with a robotic sample-handling system (Rohasys) as described previously (Van Erp et al., 2014) and the percentage oil content was normalised to 9% moisture. However, moisture content of all seed batches was within the range of 4.8–5.1%.

### 2.7 Germination

Germination tests were performed on seed batches by plating ∼65 seeds on moist filter paper in 9 cm diameter petri dishes and placing them in a growth chamber set to 20 °C and constant light (∼100 μmol m^2^ s^1^). Germination (radicle emergence) was scored visually every few hours for up to 25 h.

### 2.8 Statistical analyses

The number of biological replicates (*n*) and the standard error (SE) of the mean or standard deviation (SD) are shown. ANOVA (one-way analysis of variance) was used to assess differences between genotypes. Following significant (*P* < 0.05) F-test results, means were compared using Tukey HSD test. These analyses were performed using GenStat (18th edition, VSN International Ltd, Hemel Hempstead, UK).

## 3 Results

### 3.1 Creation of transgenic *C. sativa* lines expressing PAD under a seed specific promoter

**Fig. 2:**
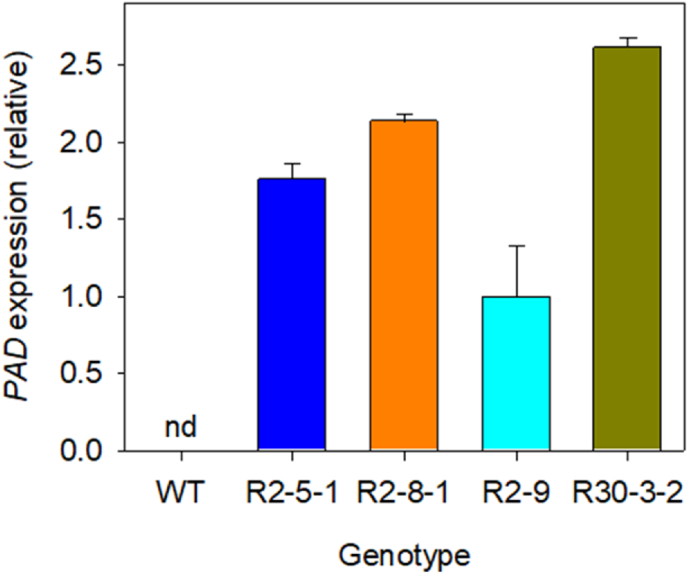
Quantitative RT-PCR analysis of *PAD* expression in seeds from *ProGLY:PAD* lines grown in the glasshouse. Values (±SE) are expressed relative to the geometric mean of the reference genes UbOxRed1 and Actin-2 (Chao et al., 2019). nd is not detected.

To express phenolic acid decarboxylase (PAD) in developing seeds of *Camelina sativa* (cv. Suneson), we synthesised a modified *B. pumilus* gene carrying an I85A substitution (Morley et al., 2013) with plant codon optimisation (Supplementary Fig. S1) and cloned it into the pBinGlyRed vector, under the control of the strong seed-specific glycinin promoter (Nguyen et al., 2013). *C. sativa* plants were transformed by the floral dip method using *Agrobacterium* (Ruiz-Lopez et al., 2014) and more than forty heterozygous T1 seeds expressing the fluorescent DsRed marker protein were selected and grown to produce segregating T2 seed. Four putative single T-DNA copy lines (R2-5-1, R2-8-1, R2-9 and R30-3-2) were selected with a 3:1 DsRed-positive seed segregation ratio and these were grown in the glasshouse to the T3 generation to produce homozygous seed batches. Expression of *PAD* in the seeds of the four homozygous lines was confirmed using quantitative RT-PCR and exhibited up to ∼2.5-fold variation, whereas no *PAD* expression was detected in wild type (WT) seeds (Fig. 2).

To determine whether this expression of *PAD* affects seed metabolite composition, as well as plant growth and development, we grew eight T4 plants of each line in individual pots in the glasshouse together with a WT control. We also planted two small (5 m^2^) plots of WT and each of the four T5 *ProGLY:PAD* lines on the experimental farm at Rothamsted Research (Harpenden, Hertfordshire, UK; grid reference TL 120130) in spring 2019.

### 3.2 Expression of PAD reduces sinapine content and produces vinyl derivatives in *C. sativa* seeds

To analyse soluble phenolic compounds in glasshouse-grown *ProGLY:PAD* seeds we prepared extracts in 80% aqueous methanol from four plants of each of the four homozygous lines plus a WT control and performed ^1^H-NMR. Sinapine was identified as the major sinapoyl ester present in WT seeds, at a level of around 7 μmol g^-1^ DW (i.e. 2.2 mg g^-1^ DW) (Table 1). In the four independent *ProGLY:PAD* lines, sinapine content was significantly (P < 0.0001) reduced by up to 95% (to around 0.5 μmol g^-1^ DW). The content of other sinapoyl esters (i.e. sinapoylglucose and sinapoylmalate) in both WT and *ProGLY:PAD* lines was below the limit of detection for quantification by ^1^H-NMR.

**Table 1:** Sinapine and 4-VP derivative content of seeds from *ProGLY:PAD* lines grown in the glasshouse and in the field.

Genotype	Sinapine	4-VP derivatives	Choline
Glasshouse			
WT	7.28 ± 0.66	nd	ND
R2-5-1	0.53 ± 0.09*	16.58 ± 0.48	ND
R2-8-1	0.68 ± 0.06*	16.11 ± 0.84	ND
R2-9	0.60 ± 0.07*	17.56 ± 0.21	ND
R30-3-2	0.56 ± 0.04*	17.46 ± 0.29	ND
Field			
WT (P1)	4.99 ± 0.23	nd	1.44 ± 0.30
WT (P2)	4.80 ± 0.06	nd	1.43 ± 0.12
R2-5-1 (P1)	0.39 ± 0.03*	15.94 ± 0.70	4.13 ± 0.33*
R2-5-1 (P2)	0.39 ± 0.03*	15.89 ± 0.69	3.56 ± 0.34*
R2-8-1 (P1)	0.35 ± 0.03*	15.00 ± 0.63	3.27 ± 0.79*
R2-8-1 (P2)	0.42 ± 0.03*	15.78 ± 1.05	3.36 ± 0.13*
R2-9 (P1)	0.42 ± 0.03*	15.76 ± 0.83	2.98 ± 0.61*
R2-9 (P2)	0.35 ± 0.03*	16.68 ± 0.27	3.46 ± 0.34*
R30-3-2 (P1)	0.35 ± 0.03*	15.11 ± 0.33	3.27 ± 0.25*
R30-3-2 (P2)	0.32 ± 0.03*	16.28 ± 0.54	2.60 ± 0.32*

Data were obtained by ^1^H-NMR analysis following extraction in CD_3_OD:D_2_O (4:1). Quantitation was carried out relative to an internal standard (3-(trimethylsilyl)propionic-2,2,3,3 acid, 0.01% w/v). Values are expressed as μmoles g^-1^ seed dry weight (DW) and are the mean ± SE of measurements made on seed batches from four plants of each genotype in the glasshouse and three seed batches from each plot for the field. ND is not determined and nd is not detected. (*ANOVA - Tukey HSD P < 0.0001).

Further examination of the ^1^H-NMR spectra of *ProGLY:PAD* lines indicated an associated increase of signals in the aromatic and olefinic region (Supplementary Fig. S2). The chemical shifts in the olefinic region were characteristic of those derived from vinyl derivatives (methenyl protons: δ 6.81–6.73 ppm (dd, *J* = 17.7, 11.0 Hz); methine protons: δ 5.88–5.74 ppm (d, *J* = 17.7 Hz) and 5.30–5.23 (d, *J* = 11.0 Hz) and indicated a family of structurally related compounds. With the additional increased signals in the aromatic region, the induced compounds were postulated to be a set of 4-VP derivatives. Quantitation, via integration of the methine signals, suggested levels of up to 17.5 μmol g^-1^ DW of this class of compounds in glasshouse-grown *ProGLY:PAD* seeds (Table 1). The molar quantity of 4-VP derivatives produced is therefore more than double that of sinapoyl esters in WT seeds. To confirm that the accumulation of 4-VP derivatives was not caused by expression of the DsRed marker protein from the pBinGlyRed vector, we also performed ^1^H-NMR analysis on a control line expressing DsRed and found that the spectra contain no chemical shifts in the olefinic regions (Supplementary Fig. S2).

^1^H-NMR analysis of 80% aqueous methanol extracts from field-grown WT and *ProGLY:PAD* seeds also showed that sinapine content was reduced by up to 95% (i.e. from 5 μmol g^-1^ DW to less than 0.4 μmol g^-1^ DW) (Table 1). We also measured the free choline content of field-grown *ProGLY:PAD* seeds and found that it was more than double that of WT (Table 1). Previous studies have shown that disruption of sinapine biosynthesis leads to accumulation of its precursor choline (Clauß et al., 2011). The sinapoylglucose and sinapoylmalate content in both WT and *ProGLY:PAD* lines were below the limit of detection, as was previously observed in glasshouse grown material. ^1^H-NMR again confirmed the presence of a family of putative 4-VP derivatives, accumulating to more than 16.7 μmol g^-1^ DW in field-grown *ProGLY:PAD* seed (Table 1).

### 3.3 PAD expression produces glycosylated 4-vinyl derivatives of multiple hydroxycinnamic acids

**Fig. 3:**
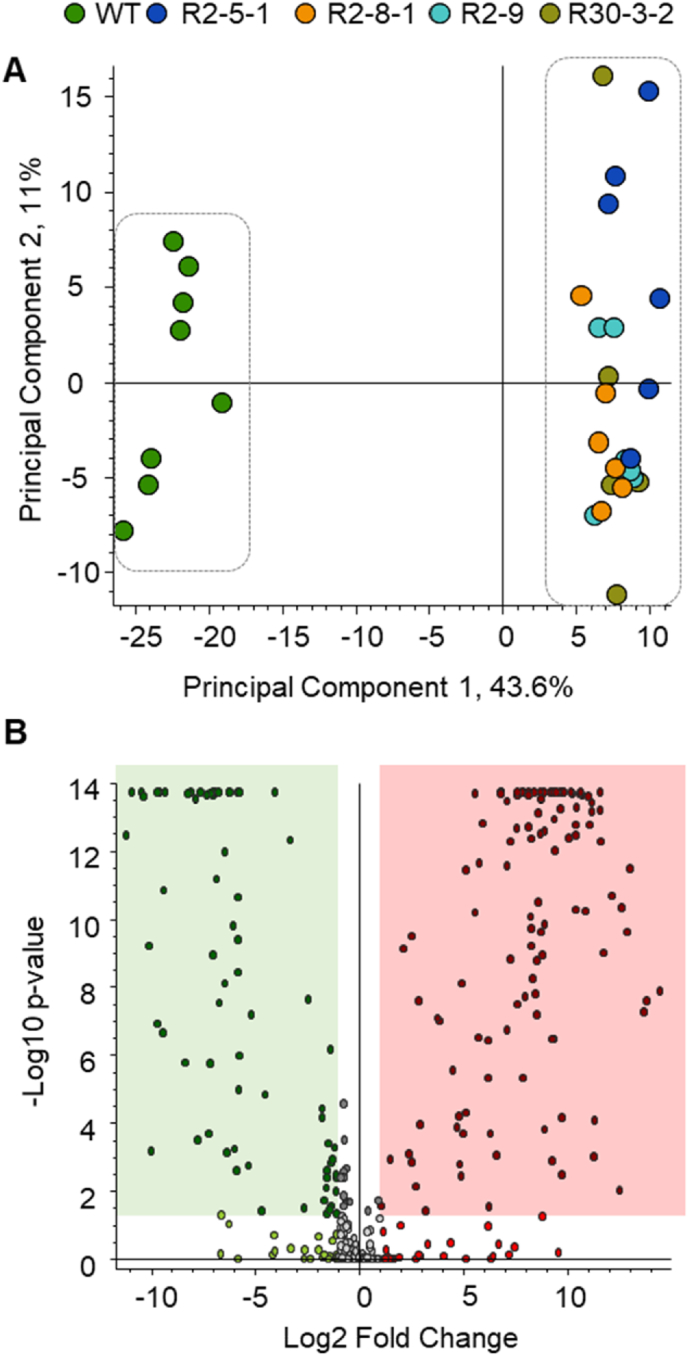
Multivariate analysis of LC-MS/MS data (negative ionisation mode) following extraction of field grown seeds of *ProGLY:PAD* lines and WT controls in aqueous methanol. A, Principal Component Analysis scores plot indicating separation of transgenic lines in the direction of PC1 (43.6%); B, Differential analysis indicating upregulated (Log2fold change >1 and P < 0.05) metabolite features in the red box and downregulated (Log2fold change < −1 and P < 0.05) in the green box. (For interpretation of the references to colour in this figure legend, the reader is referred to the Web version of this article.)

To further characterise the metabolomic changes in field-grown *ProGLY:PAD* seeds we analysed polar and non-polar extracts using ^1^H-NMR and LC-MS/MS. An unbiased metabolomic analysis of the *ProGLY:PAD* seed in negative ionisation mode high resolution LC-MS/MS showed that each of the four independent homozygous *ProGLY:PAD* lines could be separated from WT (Fig. 3A; Supplementary Fig. S3) and a differential analysis of LC-MS/MS data (Fig. 3B) revealed 119 statistically significant (P < 0.05) upregulated (Log2 fold change >1) metabolite features. After manual curation to remove isotope peaks adducts and fragment ions, 47 upregulated metabolite features explained the difference between WT and *ProGLY:PAD* seeds (Supplementary Table 2). Inspection of the accurate mass data and characteristic MS/MS fragments suggested a suite of differentially substituted 4-VP derivatives, which are essentially absent from WT *C. sativa* seeds (Supplementary Tables 2 and 3).

Abundant 4-VP derivatives were isolated by HPLC from seed of one of the *ProGLY:PAD* lines and their structures were confirmed by ^1^H and, where possible, 2D-NMR experiments. The isolated compounds comprised ten styrene metabolites and included 3,4,5-trihydroxystyrene-diglucopyranoside, 4-VP-diglucopyranoside, malonyl-3,4,5-trihydroxystyrene-diglucopyranoside, 4-VP-rhamnopyranosyl-glucopyranoside, 4-VP-apiofuranosyl-glucopyranoside, 4-VG-rhamnopyranosyl-glucopyranoside, 4-VP-(5ʹ-O-sulfonyl, 6ʹ-O-malonyl-β-glucopyranoside, 4-VP-apiofuranosyl-(6′-malonyl)-glucopyranoside, 4-VP-rhamnopyranosyl-(sulfonyl, malonyl)-glucopyranoside and 4-VP-rhamnopyranosyl-(6′-malonyl)-glucopyranoside. The presence of diglycosidic, sulfonated and malonated derivatives among the abundant isolated compounds also provided supporting evidence (characteristic MS fragments and NMR chemical shifts) for the putative assignment of a greater number of similarly substituted 4-VP analogues from the LC-MS/MS dataset (Supplementary Tables 2 and 3).

**Fig. 4:**
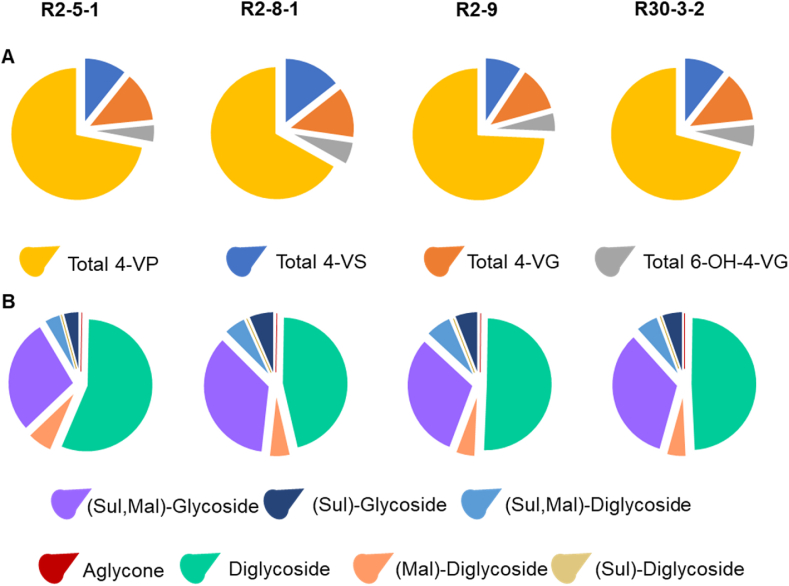
Compositional analysis of styrene analogues in seeds from field grown *ProGLY:PAD* lines. A, Comparison of aromatic substitution patterns; B, Comparison of substituent types. Data derived from curated LC-MS/MS peak area data.

A full compositional analysis indicated that each of the four independent *ProGLY:PAD* lines exhibited similar pattern of styrene derived analogues (Supplementary Table 4 and Fig. 4). By far the largest and most abundant group of induced compounds were derived from 4-VP (comprising around 70% of the total) with derivatives of 4-VS, 4-VG and 6-Hydroxy-4-VG making up a smaller component of the total styrene mix. Diglycosidic entities made up the largest proportion of the compounds in the 4-VP and 4-VG mixtures and of these the rhamnosyl-glucoside analogues dominated over apiosyl-glucoside and diglucoside entities. In 6-hydroxy-4-VG and 4-VS categories, diglycosides were not observed and the major compounds here were monoglucosylated derivatives bearing malonyl and sulfonyl substituents. We were also able to detect a significant (P < 0.0001) increase in 4-VP and 4-VS (a Log2 fold change of >8) using LC-MS/MS, but the abundance of these compounds was relatively low compared to 4-VP glycosides (Supplementary Table 4).

### 3.4 PAD expression leads to the downregulation of additional phenylpropanoid derivatives

Unbiased metabolomic analysis of *ProGLY:PAD* seeds by high resolution LC-MS/MS showed statistically significant (P < 0.05) downregulated (Log2 fold change >1) metabolite features as well as upregulated features (Supplementary Table 2; Fig. 3B). The most abundant of these downregulated metabolites is sinapine, which we quantified with ^1^H-NMR as ∼5 μmol g^-1^ DW in WT field-grown seeds (Table 1). Other metabolite features that are reduced in *ProGLY:PAD* seeds versus WT are much less abundant than sinapine (Supplementary Table 2). Inspection of the accurate mass data and characteristic MS/MS fragments suggest they include a range of phenylpropanoid derived metabolites, including those relating to sinapoyl derivatives (Supplementary Table 2). This was evident from the MS/MS analysis where ions at m/z 223 (C_11_H_11_O_5_) were detected. Although not all identities could be discerned from MS alone, several are sinapoyl glycosides (Supplementary Table 2). There was also evidence of similar feruloyl glycosides present in the set of downregulated metabolite features (Supplementary Table 2).

### 3.5 PAD expression does not adversely affect seed quality and yield

To determine whether expression of PAD in *C. sativa* affects seed yield and quality under glasshouse growth conditions, we analysed eight plants of each genotype grown in individual pots by measuring thousand grain weight (TGW), percentage oil content and seed yield per plant (Table 2). Under the growth conditions used for this experiment the TGW for WT *C. sativa* (cv Suneson) was ∼ 1g (i.e. 1 mg per seed), the oil content of the seeds was ∼34% (@ 9% moisture) and each plant yielded ∼8g of seeds. Among WT and the four independent homozygous *ProGLY:PAD* lines that we selected, no significant difference (P > 0.05) was detected in TGW, oil content or seed yield (Table 2). Germination tests were also performed on seeds of each genotype and no significant (P > 0.05) difference was observed. In all cases more than ∼98% germinated after 24 h of imbibition at 20 °C (Table 2).

**Table 2:** Seed quality and yield measurements from *ProGLY:PAD* lines grown in the glasshouse and in the field.

Genotype	TGW (g)	Oil content (%)	Seed yield (g plant^-1^/g m^2^)	Harvest index (%)	Germination (%)
Glasshouse					
WT	0.95 ± 0.06	34.32 ± 0.36	8.22 ± 0.45	ND	98.0 ± 1.2
R2-5-1	0.95 ± 0.07	34.51 ± 0.40	8.19 ± 0.79	ND	99.7 ± 0.3
R2-8-1	1.02 ± 0.09	34.70 ± 0.35	8.57 ± 0.68	ND	98.0 ± 1.5
R2-9	0.83 ± 0.13	34.04 ± 0.18	6.91 ± 0.57	ND	97.7 ± 1.3
R30-3-2	0.77 ± 0.08	33.91 ± 0.27	7.53 ± 0.93	ND	98.3 ± 1.7
Field					
WT (P1)	0.81 ± 0.03	34.02 ± 0.10	162.99	27.77	97.6 ± 1.5
WT (P2)	0.81 ± 0.01	34.69 ± 0.06	197.05	32.15	99.2 ± 0.8
R2-5-1 (P1)	0.74 ± 0.09	33.86 ± 0.02	212.84	36.25	100
R2-5-1 (P2)	0.80 ± 0.04	34.57 ± 0.04	191.69	32.58	100
R2-8-1 (P1)	0.73 ± 0.04	33.82 ± 0.10	173.42	32.32	99.4 ± 0.6
R2-8-1 (P2)	0.77 ± 0.01	34.35 ± 0.01	200.83	34.98	97.2 ± 1.8
R2-9 (P1)	0.77 ± 0.02	33.22 ± 0.09	211.14	38.19	98.3 ± 1.7
R2-9 (P2)	0.75 ± 0.04	33.45 ± 0.19	147.32	33.66	99.6 ± 0.4
R30-3-2 (P1)	0.74 ± 0.02	34.06 ± 0.12	228.24	40.27	98.6 ± 1.4
R30-3-2 (P2)	0.73 ± 0.03	33.76 ± 0.01	198.54	34.15	100

For the glasshouse, values are the mean ± SE of measurements made on seed batches from either four or eight plants of each genotype. For the field, values for thousand grain weight (TGW), oil content and germination are the mean ± SE of measurements made on three seed batches from each plot and values for harvest index and yield (in g m^2^) were determined from a 1 m^2^ area section of each plot. ND is not determined. ANOVA (P > 0.05).

To determine whether expression of PAD in *C. sativa* affects seed yield and quality under field conditions, we measured TGW, percentage oil content, harvest index and seed yield per unit area from WT and each of the four independent homozygous *ProGLY:PAD* lines that were grown on our experimental plots (Table 2). TGW for WT *C. sativa* was ∼0.8g (i.e. 0.8 mg per seed), the oil content of the seeds was ∼34% (@ 9% moisture), harvest index was ∼30% and yield ∼180 g m^2^ (extrapolates to ∼1.8 tons hectar^-1^). Among WT and the four independent homozygous *ProGLY:PAD* lines, no significant (P > 0.05) difference in in TGW, oil content, harvest index or seed yield, was detected (Table 2). The morphology of seeds collected from the field appeared to be normal for all genotypes (Supplementary Fig. 4) and germination tests showed no significant (P > 0.05) differences. In all cases more than ∼95% germinated after 24 h of imbibition at 20 °C (Table 2).

## 4 Discussion

In this study we show that sinapine (and total sinapoyl ester) content of *C. sativa* seeds can be reduced by more than 95% in both glasshouse and field by seed-specific heterologous expression of a bacterial PAD, and that the seeds instead accumulate 4-VPs, mainly in the form of mono and diglycosylated analogues (Supplementary Fig. 5). The molar quantity of these 4-VP glycosides is more than twice that of sinapoyl esters in WT seeds, suggesting that phenylpropanoid pathway flux is enhanced in response to demand by PAD, and/or that this flux is also drawn from other metabolic fates than sinapine. Previous studies have shown that disruption of the committed sinapoyl ester biosynthesis enzyme SGT, can reduce sinapine and total sinapoyl-ester content in rapeseed by up to 71% and 76%, respectively (Emrani et al., 2015; Wolfram et al., 2010). Clauβ et al. (2011) showed that sinapine esterase (SCE) overexpression can also deplete rapeseed sinapine content by ∼95%, but this does not reduce the level of other (residual) sinapoyl esters, which can account for up to a third of the total. Clauβ et al. (2011) also reported that SCE overexpression resulted in changes in seed size, water content and morphology. Our analysis of multiple *C. sativa ProGLY:PAD* lines suggests that PAD expression does not adversely affect seed weight, morphology, moisture or oil content, germination or yield under both the glasshouse and field conditions used in our study.

We proposed that by introducing PAD activity into developing embryos of *C. sativa*, multiple hydroxycinnamic acid intermediates within the phenylpropanoid pathway would be converted to their 4-vinyl derivatives, thereby depriving sinapine biosynthesis of precursors. Consistent with this hypothesis, we detected a substantial increase in the abundance of 4-VP and 4-VS, which are immediate 4-vinyl derivatives of 4-coumaric acid and sinapic acid, respectively (Fig. 1). No vinyl derivatives of caffeic acid were detected, and this is consistent with phenylpropanoid pathway flux proceeding via caffeoyl-CoA (Milkowski and Strack, 2010). We also detected a range of mono and diglycosylated 4-VPs, often also decorated with malonyl and/or sulphonyl groups. The most abundant of these compounds are derived from 4-VP (i.e. 4-VP diglucoside, 4VP rhamnosyl glucopyranoside and 4-VP apiofuranosyl glucopyranoside). Of these, 4-VP rhamnosyl glucoside is the most abundant styrene analogue we observed, an arrangement previously reported in other styrene analogues identified in bracken fern (*Pteridium aquilinum*) and maidenhair spleenwort (*Asplenium trichomanes*) (Ojika et al., 1987; Revoltella et al., 2019). Phenolic compounds are frequently glycosylated in plants (Le Roy et al., 2016) and pathway manipulation can lead to the production of new glycosides that are formed by the activities of native UTP-glycosyltransferases (UGTs). For example, Meiβner et al. (2008) showed that *Arabidopsis thaliana* seeds deficient in the SGT gene *UGT84A2* accumulate sinapoyl-4-O-hexoside. Developing *A. thaliana* seeds are known to express multiple UGTs, including those responsible for synthesising glycosylated flavonols (Jones et al., 2003; Routaboul et al., 2006; Tohge et al., 2005; Yonekura-Sakakibara et al., 2007). It is possible that the glycosylated 4-VPs present in *C. sativa ProGLY:PAD* seeds are synthesised by similar UGTs. We also found that sinapoyl (and feruloyl) glycosides are among the low abundance metabolites that are downregulated in *ProGLY:PAD* seeds and this might result from UGT substrate competition.

The only previous study we know of where flux to sinapine has been diverted to produce another compound in Brassicaceous oilseeds is Hüsken et al. (2005). In this study 4-coumaroyl-CoA was used to synthesise resveratrol glucoside (piceid) in *B. napus*. Expression of a stilbene synthase from *V. vinifera* combined with SGT RNA-interference was required to produce around 1.6 μmol g^-1^ DW of piceid (Hüsken et al., 2005). By comparison, we were able to produce ten times more 4-VP glycosides (17 μmol g^-1^ DW) in *C. sativa* seeds using expression of PAD alone. The greater production of 4-VP may be due in part to the ability of PAD to produce vinyl derivatives of multiple free hydroxycinnamic acid intermediates (Morley et al., 2013), whereas stilbene synthase requires not only 4-coumaroyl-CoA but also three molecules of malonyl-CoA to produce resveratrol (Hüsken et al., 2005). It is noteworthy that *B. napus* seeds produce several times more sinapoyl esters than *C. sativa* (Matthäus and Zubr, 2000; Zum Felde et al., 2007). Therefore, PAD expression in *B. napus* may lead to higher yields of 4-VPs than we have reported here in *C. sativa*.

4-VP glycosides are the predominant products of PAD expression in *C. sativa*. 4-VP is a building block for a range of synthetic polymers that are used in resins, inks, elastomers, and coatings (Qi et al., 2007). For example, PVP is important in electronics, where it is used as a photoresist and as a dielectric layer, for example in organic transistors of organic thin-film-transistor liquid–crystal displays (Qi et al., 2007). 4-VP is currently produced from petroleum-based feedstock, but studies have previously investigated more sustainable production routes for production of 4-VP based on fermentation of genetically engineered microorganisms (Robinson et al., 2020; Huccetogullari et al., 2019; Qi et al., 2007; Verhoef et al., 2009). Two-phase fermentation appears necessary for bacterial production of 4-VP, owing to product toxicity (Qi et al., 2007; Verhoef et al., 2009). Verhoef et al. (2009) engineered a solvent-tolerant *Pseudomonas putida* strain to produce 4-VP and showed that a second phase of 1-decanol can be used to extract 4-VP during glucose fed-batch cultivation. A maximum productivity of 0.75 mM h^−1^ and a final total 4-VP concentration of 21 mM (and 147 mM in the 1-decanol phase) were reported (Verhoef et al., 2009). Single-step bioconversion of 4-coumarate to 4-VP has also been demonstrated, with Rodriguez et al. (2021) reporting that a *Corynebacterium glutamicum* strain expressing PAD can produce 1.5 M 4-VP in the undecanol phase following batch cultivation. Much higher concentrations of 4-VP can therefore be produced using microbial fermentation than we report here as glycosides in *C. sativa* seeds. However, one possible advantage that 4-VP production in Brassicaceous oilseeds holds over microbial fermentation is that the product is derived from photosynthesis and could serve as a value-added co-product of an existing biorefining process.

4-VP glycosides have previously been detected in ferns (Ojika et al., 1987; Revoltella et al., 2019). However, they have not been reported previously in angiosperms (to our knowledge) and we show that 4-VP (and other vinyl derivatives of hydroxycinnamic acids such as 4-VS) can be made in angiosperms using metabolic engineering. There is potential to further tailor the quantity and composition of 4-VPs that can be produced in Brassicaceous oilseeds by manipulating PAD substrate specificity, phenylpropanoid pathway flux and UGT activities. Seed-specific expression of PAD may also affect the abundance of other downstream metabolites of 4-coumaric acid such as flavonoids, monolignols, stilbenes, and coumarins (Fraser and Chapple, 2011). However, our data suggest that seed development and germination are not adversely affected in *C. sativa*. Finally, given our findings, it may also be possible to produce more substantial amounts of 4-VPs in fruits such as tomato (*Solanum lycopersicum*). Zhang et al. (2015) showed that fruit-specific expression of a transcriptional regulator of flavonol biosynthesis from *A. thaliana* called MYB12, resulted in as much as 10% of tomato fruit DW accumulating as flavonols and hydroxycinnamates.

## Authorship contributions

G.N.M. conceived the original research plans, G.N.M. and P.J.E. designed and supervised the experiments; G.N.M. made the transgenics; G.N.M., M.L., and S.K. measured seed size, yield, germination, and oil content and analysed the data; R.K.B. and A.R.S. carried out gene expression analysis. C.L. carried out metabolite extractions and collected the LC-MS/MS data. J.L.W. analysed the ^1^H-NMR and LC-MS/MS metabolite data. C.N-D. isolated the vinyl phenol derivatives and confirmed their structure; P.J.E. wrote the article together with contributions from G.N.M., S.K., J.L.W. and C.N-D.

## Funding

This work was supported by the 10.13039/501100000268UK Biotechnology and Biological Sciences Research Council through grant BBS/E/C/000I0420 and BBS/E/C/000I0410 and the 10.13039/501100006324HVCfP NIBB (POC–NOV16-01).

## Declaration of competing interest

The authors declare there is no conflict of interest.
