# Supplementary material for: Diverting phenylpropanoid pathway flux from sinapine to produce industrially useful 4-vinyl derivatives of hydroxycinnamic acids in Brassicaceous oilseeds
Source: Metab Eng. 2022 Mar;70:196–205. doi: 10.1016/j.ymben.2022.01.016 (PMC8860379; doi:10.1016/j.ymben.2022.01.016)
Supplement: Multimedia component 1 [file mmc1.docx]

**Supplementary data.**

GAATTCATGGATCAATTCGTTGGATTGCATATGATCTACACTTACGAGAACGGTTGGGAGTACGAGATTTACATCAAGAACGATCATACTATTGATTACAGAATCCATTCAGGAATGGTTGGAGGTAGATGGGTGAGGGATCAGGAGGTGAACATCGTGAAGCTTACTAAGGGAGTGTACAAGGTGTCTTGGACTGAGCCTACTGGAACTGATGTGTCTTTGAACTTCATGCCTGAGGAGAAGAGGATGCATGGTGTGGCTTTCTTCCCTAAGTGGGTGCATGAGAGGCCTGACATCACTGTTTGCTACCAGAACGATTACATTGATCTTATGAAGGAGTCTAGAGAGAAGTATGAGACTTACCCTAAGTACGTGGTGCCTGAGTTCGCTGACATCACTTACATCCACCACGCTGGAGTGAACGATGAGACTATCATCGCTGAGGCACCTTACGAGGGAATGACTGATGAGATCAGGGCTGGAAGAAAGTGACCGCTCGAG

**Supplementary Fig. S1. DNA sequence of *B. pumilus* PAD gene codon optimised for expression in *C. sativa*.**

**
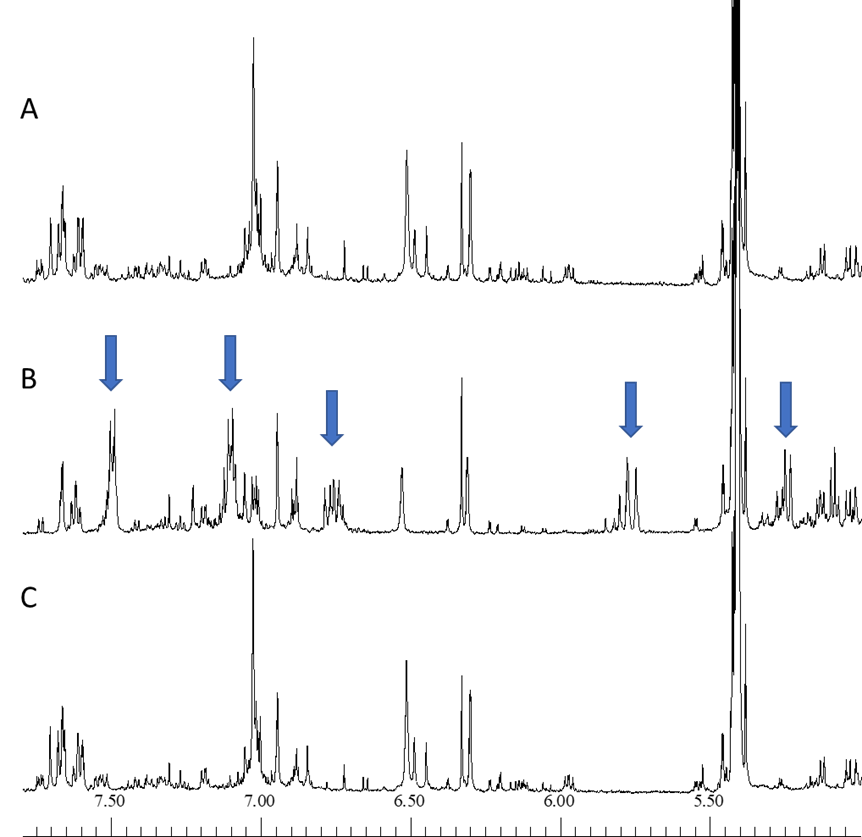
**

**Supplementary Fig. S2. Comparison of ^1^H NMR spectra (δ7.8-5.0) for WT (A), *ProGLY:PAD* (B) and DsRed (C) seed extracts indicating differences in aromatic and olefinic spectral regions.** Peaks indicated with blue arrows are consistent with those derived from phenolstyrene analogues.


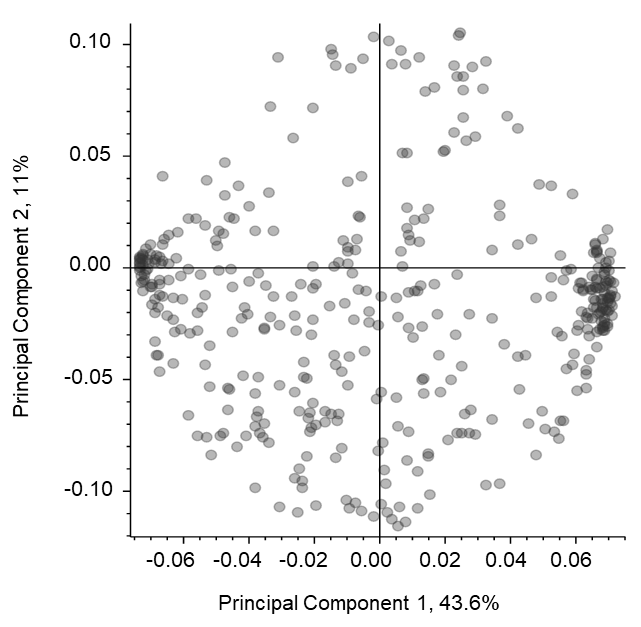


**Supplementary Fig. S3. Principal Component Analysis loadings plot for multivariate analysis of LC-MS/MS data shown in Fig. 2.**


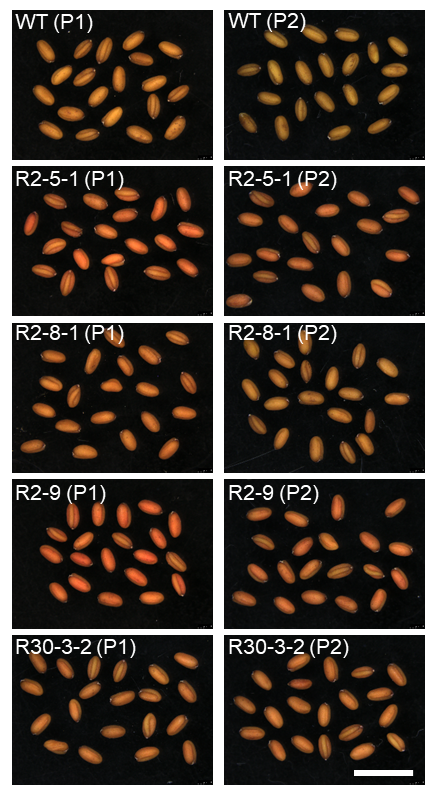


**Supplementary Fig. S4. Seed morphology of *ProGLY:PAD* lines grown in the field.** Scale bar = 5 mm.

**
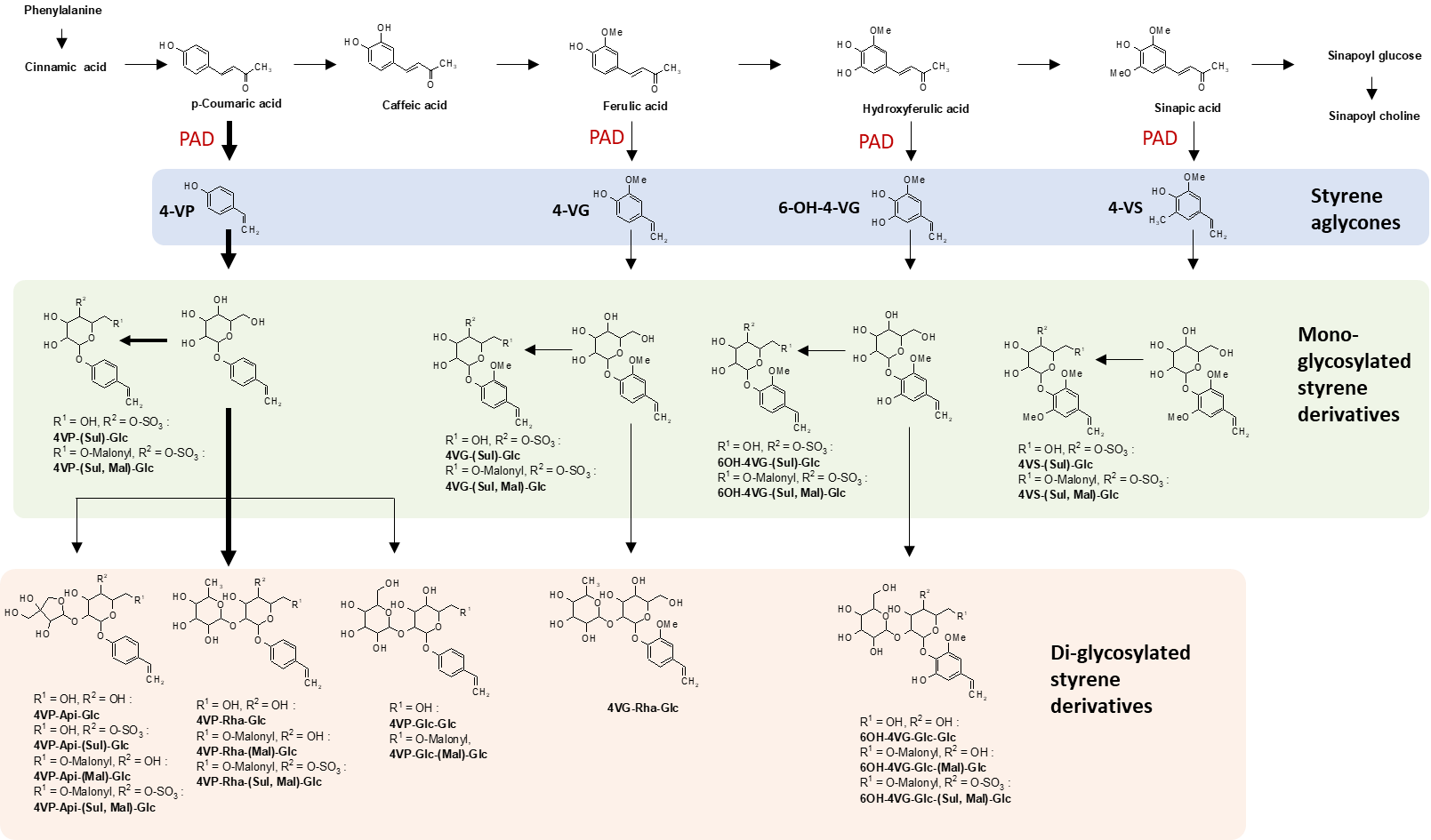
**

**Supplementary Fig. 5. Pathway illustrating diversity and biosynthetic origin of styrene analogues formed in *ProGLY:PAD* seeds.**
