# Supplementary material for: Diverting phenylpropanoid pathway flux from sinapine to produce industrially useful 4-vinyl derivatives of hydroxycinnamic acids in Brassicaceous oilseeds
Source: Metab Eng. 2022 Mar;70:196–205. doi: 10.1016/j.ymben.2022.01.016 (PMC8860379; doi:10.1016/j.ymben.2022.01.016)
Supplement: Multimedia component 2 [file mmc2.docx]

**Supplementary Table 1. Primers used in this study.**

PAD-F: CGGAATTCATGGATCAATTCGTTGGATTGCATATGA

PAD-R: CTCGAGCGGTCACTTTCTTCCAGCCCTGATCTC

UboxRed-F1: AAGGTCTCGGCGGTTTCAT

UboxRed-R1: ACGATACTTGGCAGCATACTCTAC

Actin-2-F1: AGCAGGAGATGGAGACCTCA

Actin-2-R1: ATCACAGCACTTGCACCAAG

PAD-F1: ATGGATCAATTCGTTGGATTGCATATGA

PAD-R1: AAAGACACATCAGTTCCAGTAGGCT
