# Supplementary material for: Diverting phenylpropanoid pathway flux from sinapine to produce industrially useful 4-vinyl derivatives of hydroxycinnamic acids in Brassicaceous oilseeds
Source: Metab Eng. 2022 Mar;70:196–205. doi: 10.1016/j.ymben.2022.01.016 (PMC8860379; doi:10.1016/j.ymben.2022.01.016)
Supplement: Multimedia component 4 [file mmc4.docx]

**Supplementary Table 3.** Identification of 4-VP, 4-VG and 4-VS metabolites and their characteristic MS fragmentation patterns and retention times.

| RT (min) | m/z | Formula | Ion | Delta (ppm) | MSMS | Name | Code | Identification |
| --- | --- | --- | --- | --- | --- | --- | --- | --- |
| 23.86 | 119.0507 | C_8_H_7_O | [M-H]^-^ | 3.88 | **93.0349** (C_6_H_5_O) | 4-Vinylphenol | 4-VP | From MSMS fragmentation |
| 20.47 | 361.0599 | C_14_H_17_O_9_S | [M-H]^-^ | 0.07 | **241.0028** (C_6_H_9_O_8_S), 152.9869 (C_3_H_5_O_5_S), 96.9607 (HO_4_S) | 4-Vinylphenol-*O*-(sulfonyl)-β-glucopyranoside | 4-VP-(Sul)-Glc | From MSMS fragmentation |
| 20.83 | 413.1439 | C_19_H_25_O_10_ | [M-H]^-^ | -3.41 | 281.1029 (C_14_H_17_O_6_), 161.0457 (C_6_H_9_O_5_), **119.0504** (C_8_H_7_O) | 4-Vinylphenol-*O*-apiofuranosyl-β-glucopyranoside | 4-VP-Api-Glc | Isolated and structure confirmed by NMR |
| 20.70 | 427.1601  473.1655 | C_20_H_27_O_10_  C_21_H_29_O_12_ | [M-H]^-^  [M+formate-H]^-^ | -1.99  -2.09 | 247.0834 (C_10_H_15_O_7_), 125.0249 (C_6_H_5_O_3_), **119.0504** (C_8_H_7_O)  307.1043 (C_12_H_19_O_9_), 247.0830 (C_10_H_15_O_7_), 125.0250 (C_6_H_5_O_3_), **119.0508** (C_8_H_7_O) | 4-Vinylphenol-*O*-rhamnosyl-β-glucopyranoside | 4-VP-Rha-Glc | Isolated and confirmed by NMR. Compares with reported structure. |
| 18.91  18.77 | 443.1555  443.1555 | C_20_H_27_O_11_  C_20_H_27_O_11_ | [M-H]^-^  [M-H]^-^ | -0.85  -0.85 | 281.1035 (C_14_H_17_O_6_), 221.0670 (C_8_H_13_O_7_), 161.0461 (C_6_H_9_O_5_), **119.0508** (C_8_H_7_O)  281.1034 (C_14_H_17_O_6_), 221.0671 (C_8_H_13_O_7_), 161.0460 (C_6_H_9_O_5_), **119.0506** (C_8_H_7_O) | 4-Vinylphenol-*O*-glucosyl-β-glucopyranoside | 4-VP-Glc-Glc | Isolated and structure confirmed by NMR. |
| 24.32 | 469.1711  513.1611 | C_22_H_29_O_11_  C_23_H_29_O_13_ | [M-CO_2_]^-^  [M-H]^-^ | -0.96  -0.45 | 427.1579 (C_20_H_27_O_10_), 281.1020 (C_14_H_17_O_6_), 205.0710 (C_8_H_13_O_6_), **119.0498** (C_8_H_7_O)  **119.0504** (C_8_H_7_O) | 4-Vinylphenol-*O*-rhamnosyl-(6'-*O*-malonyl)-β-glucopyranoside fragment [M-CO2] | 4-VP-(Mal)-Rha-Glc | Isolated and structure confirmed by NMR. |
| 23.11 | 447.0599 | C_17_H_19_O_12_S | [M-H]^-^ | -0.74 | 403.0695 (C_16_H_19_O_10_S), **361.0591** (C_14_H_17_O_9_S), 283.0126 (C_8_H_H_O_9_S), 241.0021 (C_6_H_9_O_8_S), 119.0505 (C_8_H_7_O), 103.0039 (C_3_H_3_O_4_), 96.9604 (HO_4_S) | 4-Vinylphenol-*O*-(5'-O-sulfonyl, 6'-*O*-malonyl)-β-glucopyranoside | 4-VP-(Sul,Mal)-Glc | Isolated and structure confirmed by NMR. |
| 22.25 | 485.1662 | C_22_H_29_O_12_ | [M-CO_2_]^-^ | -0.51 | 443.1552 (C_20_H_27_O_11_), 281.1033 (C_14_H_17_O_6_), 221.0668 (C_8_H_13_O_7_), 161.0458 (C_6_H_9_O_5_), **119.0505** (C_8_H_7_O) | 4-Vinylphenol-*O*-(malonylglucosyl)-β-glucopyranoside | 4-VP-(Mal-Glc)-Glc | From MSMS fragmentation |
| 21.27 | 493.1020 | C_19_H_25_O_13_S | [M-H]^-^ | -0.35 | **373.0442** (C_11_H_17_O_12_S), 293.0880 (C_11_H_17_O_9_), 239.9947 (C_6_H_8_O_8_S), 180.9814 (C_4_H_5_O_6_S), 152.9867 (C_3_H_7_O), 96.9604 (HO_4_S) | 4-Vinylphenol-*O*-apiofuranosyl-β-(sulfonyl)-β-glucopyranoside | 4-VP-Api-(Sul)-Glc | From MSMS fragmentation |
| 23.88 | 499.1458 | C_22_H_27_O_13_ | [M-H]^-^ | 0.15 | 455.1539 (C_21_H_27_O_11_), 413.1444 (C_19_H_25_O_10_), 161.0446 (C_6_H_9_O_5_), **119.0505** (C_8_H_7_O) | 4-Vinylphenol-*O*-apiofuranosyl-(6’-*O*-malonyl)-β-glucopyranoside | 4-VP-Api-(Mal)-Glc | Isolated and structure confirmed by NMR. |
| 23.35 | 579.1016 | C_22_H_27_O_16_S | [M-H]^-^ | -1.66 | 535.1119 (C_21_H_27_O_14_S), **493.1020** (C_19_H_25_O_13_S), 475.0920 (C_19_H_23_O_12_S), 415.0549 (C_13_H_19_O_13_S), 373.0450 (C_11_H_17_O_12_S), 119.0507 (C_8_H_7_O), 96.9606 (HO_4_S) | 4-Vinylphenol-*O*-apiofuranosyl-(sulfonyl, malonyl)-β-glucopyranoside | 4-VP-Api-(Sul,Mal)-Glc | From MSMS fragmentation |
| 23.56 | 593.1171 | C_23_H_29_O_16_S | [M-H]^-^ | -1.83 | 549.1278 (C_22_H_29_O_14_S), **507.1168** (C_20_H_27_O_13_S), 489.1076 (C_20_H_25_O_12_S), 429.0700 (C_14_H_21_O_13_S), 387.0602 (C_12_H_19_O_12_S), 119.0506 (C_8_H_7_O), 96.9605 (HO_4_S) | 4-Vinylphenol-*O*-(sulfonyl, malonyl)-rhamnosyl-β-glucopyranoside | 4-VP-(Sul,Mal)-Rha-Glc | Isolated and structure confirmed by NMR |
| 21.77 | 391.0703 | C_15_H_19_O_10_S | [M-H]^-^ | -0.26 | **241.0022** (C_6_H_9_O_8_S), 96.9605 (HO_4_S) | 4-Vinylguaiacol-1-*O*-β-(sulfonyl)-β-glucopyranoside isomer | 4-VG-(Sul)-Glc | From MSMS fragmentation. Known compound. |
| 23.72 | 477.0703 | C_18_H_21_O_13_S | [M-H]^-^ | -1.02 | 433.0808 (C_17_H_21_O_11_S), **391.0705** (C_15_H_19_O_10_S), 283.0130 (C_8_H_H_O_9_S), 241.0021 (C_6_H_9_O_8_S), 149.0612 (C_9_H_9_O_2_), 96.9605 (HO_4_S) | 4-Vinylguaiacol-1-*O*-(sulfonyl, malonyl)-β-glucopyranoside | 4-VG-(Sul,Mal)-Glc | From MSMS fragmentation |
| 21.47 | 503.1763  457.1715 | C_22_H_31_O_13_  C_21_H_29_O_11_ | [M+formate-H]^-^  [M-H]^-^ | -1.46  -0.08 | 307.1040 (C_12_H_19_O_9_), 247.0828 (C_10_H_15_O_7_), 163.0618 (C_6_H_11_O_5_), **149.0613** (C_9_H_9_O_2_) | 4-Vinylguaiacol-1-*O*-rhamnosyl-β-glucopyranoside | 4-VG-Rha-Glc | Isolated and structure confirmed by NMR. |
| 21.63 | 407.0652 | C_15_H_19_O_11_S | [M-H]^-^ | -0.28 | **241.0024** (C_6_H_9_O_8_S), 165.0565 (C_9_H_0_O_3_), 96.9606 (HO_4_S) | 6-Hydroxy-4-Vinylguaiacol-*O*-(sulfonyl)-glucopyranoside | 6-OH-4-VG-(Sul)-Glc | From MSMS fragmentation |
| 23.03 | 493.0634 | C_18_H_21_O_14_S | [M-H]^-^ | -4.74 | 407.0644 (C_15_H_19_O_11_S), **283.0125** (C_8_H_11_O_9_S), 241.0020 (C_6_H_9_O_8_S), 165.0558 (C_9_H_9_O_3_), 96.9603 (HO_4_S) | 6-Hydroxy-4-Vinylguaiacol-1-*O*-(sulfonyl, malonyl)-β-glucopyranoside | 6-OH-4-VG-(Sul,Mal)-Glc | From MSMS fragmentation |
| 19.58 | 575.1610 | C_24_H_31_O_16_ | [M-H]^-^ | -1.27 | **165.0556** (C_9_H_9_O_3_) | 6-Hydroxy-4-Vinylguaiacol-(malonyl)-diglucopyranoside | 6-OH-4-VG-(Mal)-Glc-Glc | From MSMS fragmentation |
| 19.05 | 655.1177 | C_24_H_31_O_19_S | [M-H]^-^ | -1.32 | 449.0572 (C_17_H_21_O_12_S), 327.1083 (C_15_H_19_O_8_), 283.0128 (C_8_H_11_O_9_S), **241.0022** (C_6_H_9_O_8_S), 165.0560 (C_9_H_9_O_3_), 96.9605 (HO_4_S) | 6-Hydroxy-4-Vinylguaiacol-(sulfonyl, malonyl)-diglucopyranoside | 6OH-4-VG-(Sul,Mal)-Glc-Glc | From MSMS fragmentation |
| 23.94 | 179.0716 | C_10_H_11_O_3_ | [M-H]^-^ | 1.52 | **164.0480** (C_9_H_8_O_3_), 149.0248 (C_8_H_5_O_3_) | 4-Vinylsyringol (Canolol) | 4-VS | From MSMS fragmentation |
| 22.32 | 421.0807 | C_16_H_21_O_11_S | [M-H]^-^ | -0.70 | **241.0022** (C_6_H_9_O_8_S), 179.0718 (C_10_H_11_O_3_), 96.9605 (HO_4_S) | 4-Vinylsyringol-1-*O*-(sulfonyl)-β-glucopyranoside | 4-VS-(Sul)-Glc | From MSMS fragmentation |
| 23.40 | 507.0799 | C_19_H_23_O_14_S | [M-H]^-^ | -3.04 | 463.0907 (C_18_H_23_O_12_S), 421.0803 (C_16_H_21_O_11_S), 283.0127 (C_8_H_11_O_9_S), **241.0021** (C_6_H_9_O_8_S), 179.0716 (C_10_H_11_O_3_), 96.9605 (HO_4_S) | 4-Vinylsyringol-1-*O-*(sulfonyl, malonyl)-β-glucopyranoside | 4-VS-(Sul,Mal)-Glc | From MSMS fragmentation |
| 16.33  16.68 | 475.1454  475.1455 | C_20_H_27_O_13_  C_20_H_27_O_13_ | [M-H]^-^  [M-H]^-^ | -0.62  -0.49 | **313.0925** (C_14_H_17_O_8_), 151.0403 (C_8_H_7_O_3_)  **313.0927** (C_14_H_17_O_8_), 151.0403 (C_8_H_7_O_3_) | 2,6-Dihydroxy-4-Vinylphenol-*O*-diglucopyranoside | 2,6-OH-4-VP-Glc-Glc | Isolated and structure confirmed by NMR. |
| 19.21 | 517.1559  561.1457 | C_22_H_29_O_14_  C_23_H_29_O_16_ | [M-CO_2_]^-^  [M-H]^-^ | -0.65  -0.65 | **355.1034** (C_16_H_19_O_9_), **313.0929** (C_14_H_17_O_8_), 151.0404 (C_8_H_7_O_3_) | 2,6-Dihydroxy-4-Vinylphenol-*O*-(malonyl)-diglucopyranoside | 2,6-OH-4-VP-(Mal)-Glc-Glc | Isolated and structure confirmed by NMR. |
