# Supplementary material for: Diverting phenylpropanoid pathway flux from sinapine to produce industrially useful 4-vinyl derivatives of hydroxycinnamic acids in Brassicaceous oilseeds
Source: Metab Eng. 2022 Mar;70:196–205. doi: 10.1016/j.ymben.2022.01.016 (PMC8860379; doi:10.1016/j.ymben.2022.01.016)
Supplement: Multimedia component 5 [file mmc5.docx]

**Supplementary Table 4. Quantified peak areas for 4-VP derivatives from LC-MS/MS analysis (negative ion mode) of field grown *ProGLY:PAD* lines.**

| Name of Metabolite | Abbreviation | WT | R2-5-1 | R2-8-1 | R2-9 | R30-3-2 |
| --- | --- | --- | --- | --- | --- | --- |
| 4-Vinylphenol | 4-VP | 0.1 ±0.04 | 22 ±1 | 16 ±2 | 18 ±4 | 17 ±3 |
| 4-Vinylphenol-*O*-rhamnosyl-glucopyranoside | 4-VP-Rha-Glc | 3 ±5 | 1571 ±152 | 1201 ±14 | 1638 ±28 | 1353 ±207 |
| 4-Vinylphenol-*O* -(5'-*O*-sulfonyl, 6'-*O*-malonyl)-β glucopyranoside | 4-VP-(Sul,Mal)-Glc | 9 ±2 | 1039 ±220 | 1222 ±177 | 1471 ±177 | 1402 ±14 |
| 4-Vinylphenol-*O*-apiofuranosyl-β-glucopyranoside | 4-VP-Api-Glc | 0.3 ±0.2 | 752 ±52 | 557 ±97 | 718 ±69 | 688 ±53 |
| 4-Vinylphenol-*O*-(sulfonyl, malonyl)-rhamnosyl glucopyranoside | 4-VP- (Sul,Mal)-Rha-Glc | 0.5 ±0.5 | 285 ±29 | 383 ±44 | 495 ±10 | 383 ±60 |
| 4-Vinylphenol-*O-*rhamnosyl-(6'-malonyl)-glucopyranoside | 4-VP-Rha-(Mal)-Glc | 1 ±1 | 360 ±149 | 289 ±30 | 299 ±78 | 290 ±54 |
| 4-Vinylphenol-*O*-(sulfonyl)-glucopyranoside | 4-VP-(Sul)-Glc | 1 ±0.3 | 115 ±87 | 226 ±25 | 258 ±92 | 163 ±122 |
| 4-Vinylphenol-*O*-apiofuranosyl-(sulfonyl, malonyl)-glucopyranoside | 4-VP-Api-(Sul,Mal)-Glc | 0.2 ±0.01 | 82 ±6 | 114 ±11 | 163 ±39 | 142 ±23 |
| 4-Vinylphenol-*O*-apiofuranosyl- (6'-malonyl)-glucopyranoside | 4-VP-Api-(Mal)-Glc | 0.1 ±0.2 | 117 ±16 | 107 ±21 | 123 ±3 | 102 ±3 |
| 4-Vinylphenol diglucoside isomer | 4-VP-Glc-Glc | 0.1 ±0.1 | 109 ±18 | 109 ±15 | 91 ±9 | 89 ±10 |
| 4-Vinylphenol-*O*-apiofuranosyl- (sulfonyl)-glucopyranoside | 4-VP-Api-(Sul)-Glc | 0.04 ±0.01 | 15 ±12 | 36 ±3 | 49 ±6 | 37 ±0.6 |
| 4-Vinylphenol diglucoside isomer - malonate | 4-VP-(Mal)-Glc-Glc | 0.1 ±0.1 | 43 ±12 | 34 ±5 | 31 ±1 | 27 ±1 |
| 4-Vinylphenol diglucoside isomer | 4-VP-Glc-Glc | 0.1 ±0.1 | 33 ±1 | 34 ±3 | 29 ±3 | 33 ±2 |
| 4-Vinylguaiacol-1-*O*-rhamnosyl-glucopyranoside | 4-VG-Rha-Glc | 16 ±26 | 981 ±44 | 939 ±202 | 9398 ±186 | 9596 ±272 |
| 4-Vinylguaiacol-1-*O*-(sulfonyl, malonyl)-glucopyranoside | 4-VG-(Sul,Mal)-Glc | 2 ±2 | 106 ±9 | 147 ±14 | 1478 ±25 | 1522 ±35 |
| 4-Vinylguaiacol-1-*O*-(sulfonyl)-glucopyranoside isomer | 4-VG-(Sul)-Glc | 0.7 ±0.1 | 18 ±9 | 28 ±0.1 | 294 ±35 | 256 ±49 |
| 6-Hydroxy-4-vinylguaiacol-1-*O*-(sulfonyl, malonyl)-glucopyranoside | 6-OH-4-VG-(Sul,Mal)-Glc | 0.3 ±1 | 351 ±40 | 431 ±53 | 412 ±8 | 447 ±23 |
| 6-Hydroxy 4-vinylguaiacol-*O*-(sulfonyl)-glucopyranoside | 6-OH-4-VG-(Sul)-Glc | 0.1 ±0.1 | 38 ±11 | 63 ±0.4 | 65 ±6 | 57 ±8 |
| 6-hydroxy-4-vinylguaiacol-*O*-(sulfonyl, malonyl)-diglucopyranoside | 6-OH-VG-(Sul,Mal)-Glc-Glc | 0.02 ±0.01 | 9 ±1 | 10 ±0.6 | 11 ±0.3 | 10 ±1 |
| 6-Hydroxy-4-vinylguaiacol-*O*-(malonyl)-diglucopyranoside | 6-OH-VG-(Mal)-Glc-Glc | 0.1 ±0.02 | 11 ±2 | 11 ±0.1 | 8 ±0.2 | 8 ±1 |
| 4-Vinylsyringol (Canolol) | 4-VS | 0.03 ±0.01 | 7 ±1 | 8 ±0.4 | 6 ±1 | 6 ±1 |
| 4-Vinylsyringol-1-*O*- (sulfonyl, malonyl)-glucopyranoside | 4-VS-(Sul,Mal)-Glc | 0.7 ±1 | 805 ±49 | 1074 ±118 | 780 ±54 | 795 ±91 |
| 4-Vinylsyringol-1-*O*-(sulfonyl)- glucopyranoside | 4-VS-(Sul)-Glc | 0.1 ±0.1 | 88 ±4 | 124 ±13 | 90 ±6 | 101 ±9 |

Values are the mean ± SD of measurements on three aqueous methanol extracts. For clarity values have been divided through by 100,000.
